# China's Hainan Free Trade Port: Medical Laws and Policy Reform

**DOI:** 10.3389/fpubh.2021.764977

**Published:** 2021-11-15

**Authors:** Fei Qi, Yuqi Wu, Jing Wang, Qi Wang

**Affiliations:** ^1^Law School, Hainan University, Haikou, China; ^2^Danzhou City Construction Investment Co., Ltd., Danzhou, China; ^3^Henan Breast Cancer Center, The Affiliated Cancer Hospital of Zhengzhou University & Henan Cancer Hospital, Zhengzhou, China

**Keywords:** Hainan free trade port, medicine and sanitary equipment, franchise, real-world data, shared hospitals

## Abstract

In 2018, the government of China decided to develop Hainan Province as the country's first free trade port operating within the country's socialist system. Based on this strategy, Hainan reformed its medical laws and policies to make it freer and more open. For example, Hainan formulated policies for more rapid and convenient access of foreign medicine and sanitary equipment (MSE), allowed manufacturers to register MSE in China with real-world data (RWD), and provided legal and visa conveniences for international medical teams to carry out various activities, including: diagnosis, treatment and scientific research. Hainan's reforms are not only conducive to the improvement of local medical and public health levels, but also provide opportunities for international MSE manufacturers and medical research institutions swiftly to enter China's huge medical market. However, with opportunity comes risk: Hainan should be on guard against public health risks associated with medical tourism, and decide how to strike a reasonable balance between protecting local MSE enterprises and improving the accessibility of imported MSE through policies and legislation. Finally, the paper recommends that Hainan should improve the regulatory system as soon as possible to ensure the quality of diagnosis and treatment in its new hospitals, and deal with data and information security risks in the RWD research.

## Introduction

China is now the world's largest exporter and commodity trader (1). As an export-oriented developing country, the main threats facing China's economy are the decline in demand from its major trading partners and the rise of trade protectionism, which lead China to continue to pursue further opening up of its domestic market and the promotion of industrial upgrading (2). In pursuit of this goal, China proposes establishment of a new free trade port to compete with Dubai and Singapore (3). As the latest experiment in China's institutional innovation, the planned free trade port will be freer and more open than existing free trade areas in the country (4).

Earlier, China had put forward two cooperation initiatives: “Silk Road Economic Belt” and “21st Century Maritime Silk Road” in 2013 (known collectively as “The Belt and Road”, B&R) (5). This new international economic cooperation framework aims to revive China's ancient trade route connecting Europe and Africa, to promote the economic growth of potential markets and improve the interconnection between China and these markets, in order to promote diversification of domestic trade and investment (6, 7). Based on the B&R initiative and the fact that more than 90% of China's foreign trade is borne by the sea, China chose Hainan Province, China's largest special economic zone, as an important node on the ancient Maritime Silk Road, and the location of the new free trade port, with the intention of making it an important hub connecting the Indian and Pacific Oceans (8, 9).

In 2018, China's President Xi Jinping announced that the country would build its first socialist system-based free trade port in Hainan Province. The aim was to develop it as a pilot zone for China's exploration of further reform and the opening-up of various fields (10). The central government has enabled the Hainan Free Trade Port (HFTP) to quickly gain a high degree of autonomy by reforming or revising relevant policies, administrative regulations and departmental rules. With autonomy, HFTP has carried out comprehensive reforms in various fields including finance, medical care, and immigration policies (3). Regarding medical issues, HFTP has issued a series of laws and policies designed to speed up the import of medicine and sanitary equipment (MSE), making foreign enterprises and social organizations significantly less restricted by policies or laws when they carry out trade and exchanges in HFTP compared to elsewhere in China (11). Finally, HFTP has promised within its jurisdiction to liberalize cutting-edge medical research (such as stem cell clinical research) (12).

It is worth noting that the reform efficiency of other free trade zones before HFTP was partly constrained by the speed with which the relevant laws were amended. This is mainly because China is a country with typical civil laws. Its legal system represents a hierarchical pyramid structure, with lower-level and local laws subservient to higher-level laws and without ability to exceed their authority (13). Revision of higher-level laws normally requires adoption by China's highest legislature, the National People's Congress (NPC), and this takes a long time (14). In order to ensure HTFP's reforms efficiency, the Standing Committee of the NPC promulgated the *Hainan Free Trade Port Law of the People's Republic of China* on June 10, 2021. The law granted Hainan Free Trade Port (HFTP) a high degree of legislative autonomy. According to Article 10 of the law, HFTP can suspend application of some national laws and regulations in the region and gained the right to formulate local laws in relation to special policies which are only applicable within the region (15). This unprecedented autonomy in legislation and policy-making is the biggest difference between HFTP and other free trade zones in China, which also provides legal protection for HFTP's reform measures in the medical field.

The reforms of HFTP are not only conducive to the improvement of medical and public health conditions in the region, but also provide guidance for China's future general reforms in related fields. This paper seeks to discuss the aspects of HFTP's reforms that have entered the practical stage and achieved certain results, to analyze the influences of the reforms on its medical and public health services, and to assess challenges linked to the reforms.

## Specific Reform Measures in HFTP

As shown in Table 1, the reform measures announced by HFTP in the field of medicine can be summarized in a framework comprising four modules: “Franchise Medical Care”, “Franchise Operation”, “Franchise International Communication”, and “Franchising in International medical exchanges”.

**Table 1:** The reform measures announced by HFTP in the field of medicine.

**Franchise Medical Care**	1. MSE that is listed abroad but not domestically can be used in a small range of medical institutions within HFTP. 2. Set up a special import MSE review scoring center in HFTP to simplify the evaluation process of its licensed imports. 3. Patients from medical institutions in HFTP can leave with a reasonable amount of licensed imported MSE. 4. The RWD generated by imported MSE in HFTP can be collected and used for medical research and MSE registration in China. 5. Members of public hospitals and medical institutions in other parts of China can be licensed to practice part-time in HFTP.
**Franchise Operation**	1. Well-known hospitals can authorize their brand, trademark, technology, management, etc. to medical institutions in HFTP for use, 2. Imported monoclonal antibodies and other low-risk special items are subject to “warehouse first, quarantine later”. 3. Remove restrictions on foreign shareholding in health insurance and life insurance companies within HFTP.
**Franchise International Communication**	1. Research on cutting-edge medical technologies such as stem cells and their transformational applications can be carried out in HFTP. 2. Overseas research institutes and medical colleges can establish branches in HFTP according to law.
**Franchising in International medical exchanges**	1. Foreign medical staff can carry out medical activities in HFTP according to law, and can apply for a work-type residence permit consistent with the working period. 2. Family members of foreign patients can apply for private affairs visas or residence permits during the patient's medical service period. 3. Overseas manufacturers can set up after-sales centers in HFTP to provide after-sales services for MSEs that are used in HFTP but have not yet been registered in China.

Due to insufficient time, some measures in the framework lack sufficiently clear practical results to test their effectiveness. In particular, travel restrictions caused by the Covid-19 pandemic brought many measures related to immigration policies, whereby “Franchising in International medical exchanges” almost came to a standstill. On the other hand, several of the reforms have already entered the practical stage and achieved certain results, including reform of optimizing MSE import mode, recognizing real-world data and attracting non-resident medical resources.

### Making MSE Import Channels More Convenient

According to Articles 35 and 36 of the Regulations for Implementation of the *Drug Administration Law of the People's Republic of China*, a drug that has been marketed in another country must be confirmed safe, effective and clinically necessary by the National Medical Products Administration (NMPA) before it can be imported (16). To complete registration, pharmaceutical companies must carry out three phases of randomized controlled trials (RCTs) for several years. This makes it very difficult for new, specific drugs from abroad to enter China promptly (11).

In addition, while small quantities of urgently needed drugs can be imported by medical institutions for clinical use under legal authorization upon the approval of the NMPA, their use is restricted to specific medical activities in designated medical institutions (16). This means that patients can only be treated with urgently needed imported drugs in a specific medical institution, and cannot remove these drugs from a specific medical institution. When using urgently needed imported drugs which are crucial to a patient's treatment, they must be hospitalized in accordance with the regime determined by the specific course of treatment. This significantly increases the duration, cost and financial burden for patients.

China's central government has authorized HFTP to independently approve the import of MSE within its region (16). As a result, HFTP allows related enterprises to freely apply to import MSE already marketed in developed countries within its region, (17). The average approval period is between three and seven days (18). Some low-risk MSE can enter a HFTP bonded warehouse prior to the declaration process (19), which reduces to one day the time from application to use of imported MSE. Additionally, HFTP is vested with the right to apply to the Ministry of Finance to adjust some MSE tariffs annually according to demand (20).

The drug ripretinib is a successful example of HFTP's reform. It is the latest specific drug for the treatment of gastrointestinal stromal tumor (GIST) (21). Annually, in China there are about 30,000 newly diagnosed GIST cases and over 100,000 GIST patients receiving treatment (22). Outside the HFTP, it can take years before the drug is allowed to be used in the treatment of patients in China. In HFTP, however, approval for small-scale use of ripretinib followed within 2 months after the drug achieved global approval in May 2020. As a result, ripretinib became the first new drug that Chinese patients could receive at the same time as patients elsewhere in the world (23). In addition, HFTP allows patients to leave medical facilities each time with drugs for one course of treatment, which effectively cuts down tariff costs (24).

### Real-World Data (RWD) Can Be Used for MSE Registration

MSE is the cornerstone for promoting good public health. Clinical evidence of evidence-based medicine, a new model of modern clinical practice and research, mainly comes from large-sample RCTs and meta-analyses (25). However, due to the current lack of sound mutual recognition mechanism for foreign RCTs, it may take years to conduct RCTs before a foreign MSE can enter the Chinese market.

RCTs are currently the recognized standard for evaluating the efficacy of MSE in countries around the world (25). RCT results are generally required for the approval of MSE registration and marketing and for the addition of indications after marketing. With the development of evidence-based medicine, however, the RCTs' limitations have gradually become clear. First, the research conclusions of RCTs may be challenged via experiences in clinical practice. Due to strict inclusion and exclusion criteria, a trial population cannot fully represent the target population, but only reflect the efficacy of MSE in specific situations. The standard intervention adopted may not be completely consistent with clinical practice. Moreover, the limited sample size and short follow-up time result may lead to underestimation of rare adverse events. Second, research resources are very limited for some rare or infrequent diseases and, as a result, it is difficult to carry out traditional RCTs. As a result, enterprises may reduce interest in research investment, considering the limited economic benefits. Third, traditional RCTs generally demand considerable investment of time and money (26).

In recent years, real-world study (RWS) has attracted increasing attention. RWS is based on the premise of non-intervention, that is, where treatment measures are non-randomly selected according to the actual condition and wishes of patients. Data related to the MSE used and the health status of patients (i.e., RWD) is then collected and analyzed. Conclusions are drawn from evidence from large samples, long-term evaluation and statistical analysis, so as to obtain data on evidence-based medicine for MSE. The purpose is to ascertain its medical effectiveness, that is, sufficient real-world evidence (RWE) (27). In 2016, the *21st Century Cures Act* was passed in the USA, which authorized the US Food and Drug Administration to use RWD, where appropriate, as the approval evidence for post-market research and new indication development of MSE (28).

Currently, RCT data are still required in Chinese law for the application of registration and marketing of MSE or the addition of indications for MSE. However, with the support of the central government, HFTP has suspended the application of the relevant provisions in the current *Regulations for the Supervision and Administration of Medical Devices in the region*. Hainan has promulgated the *Implementation Plan of the Pilot Work of Clinical Real-World Data Application in Hainan Boao-Lecheng Pilot Zone of International Medical Tourism*. According to this policy, MSE without registration in China can be imported and used on a small scale in the region after being licensed. The clinical data generated during the use of licensed MSE can be converted into RWD and RWE for registration and approval in China after scientific and strict design, collection, processing, analysis and evaluation (29).

The above reform not only offers a legal way for Chinese patients to quickly obtain imported MSE, but also significantly increases the speed of foreign MSE entering the Chinese market. For example, after Allergan obtained RWD for its product “XEN Glaucoma Treatment System” in HFTP, the RWE acquired by the analysis successfully passed the marketing approval of NMPA. The process took fewer than 5 months overall, while the product registration cycle was seven times shorter (30).

### Attracting Non-resident Medical Resources Through “Shared Hospitals”

As the only tropical island province in China, Hainan has long been famous for tourism not its medical industry. Once HFTP's medical and public health resources were chronically inadequate. For example, in July 2018, Hainan, with a resident population of about 9.257 million, had only 1,094 general practitioners, that is, an average of only 1.18 general practitioners for every 10,000 people (31).

Moreover, restricted by China's relatively strict physician management system, it was difficult for HFTP to gain much in the way of physician resources in a short period. Currently, the practice qualification of physicians is tied to their medical institutions. In the past, physicians in some economically developed areas with abundant medical resources in China (such as Shanghai) were allowed to travel to other regions for treatment on a personal basis. However, the central government discovered that this regime of diagnosis and treatment bred medical corruption. For example, some physicians began to neglect their duties and sought to practice medicine under their own names so as to generate higher incomes (32). In response, in 2005 the Chinese government promulgated the Interim Provisions on the Management of Physicians' Outing Consultation. The aim was to prohibit private diagnosis and treatment. According to the Provisions, physicians are not allowed to carry out medical activities under their personal name, while cross-regional and cross-institutional diagnosis and treatment can only be undertaken with the agreement of relevant institutions (33). Income from such diagnosis and treatment cannot be paid to the physician directly, but is realized by the hospital where he or she practices via a reasonable increase in wages during the same period (33).

In response, HFTP experimented in becoming a platform provider which “does not ask for ownership, but for use” rather than a resource owner. HFTP also established a new type of “shared hospital” (34), responsible for formulation of a management system, daily supervision, MSE procurement and other management and operation activities. Medical teams from outside the HFTP region can be stationed in the hospital as specialists. They are not required to be resident in Hainan and are able to conduct diagnosis and treatment in the hospital when there is a medical need. All medical teams have equal paid sharing of hospitals' imaging diagnosis centers, surgery centers, hospital beds and other resources. Financial settlement is at their own risk. The MSE needed for diagnosis and treatment, but without registration in China, can be acquired through licensed imports. Benefiting from HFTP's liberal immigration policy, medical teams from other countries and regions other than the Chinese mainland can also apply for admission to the hospital for business reasons.

## Discussion

In general, HFTP has quickly carried out a large number of reforms in the medical field, providing increased convenience and preferential policies for medical resources from other regions or countries to enter China. This has also had positive impacts on the public health status of HFTP. On the other hand, HFTP's reforms also face significant challenges.

### HFTP's Medical Laws and Policy Reforms: Positive Outcomes

HFTP has limited local medical resources with a per capita gross domestic product of fewer than 10,000 US dollars (35). This makes it in the short term difficult to invest heavily in health care and public health. As a result, carrying out institutional reforms to encourage entry of extra-regional capital and medical resources is a realistic option for HFTP. The current effect is that the greatest significance of the reforms is to make HFTP the most open region for the Chinese mainland to import MSE and foreign medical teams.

Such openness offers HFTP residents the opportunity to enjoy the latest MSE in line with the international market and the expertise and resources of advanced medical teams from other countries and regions. Due to the high-level of tariff autonomy in HFTP and the good quality and low cost of medical infrastructure in the region, the prices of related MSE and diagnosis and treatment services has been significantly reduced, effectively lowering patients' treatment costs. For example, the cost of Boston keratoprothesis in HFTP is roughly 14% of that of similar operations abroad (36).

HFTP's openness is also conducive to the improvement of the health status of some disabled people. For example, the specific MSE urgently needed by some disabled people with rare diseases (such as multiple sclerosis) is not produced in the Chinese mainland. To enter China's market (37), this type of foreign MSE must go through three phases of RCTs. As a result, it is difficult for affected disabled people to obtain such medication. However, the open and more human-oriented medical policies of the HFTP greatly reduce the time and economic costs for the above-mentioned MSE to enter HFTP. This increases the accessibility of the latest international MSE in the region (38). Considering that disabled people are often economically poorer than healthy people (13), then reducing their rehabilitation costs helps to achieve greater health equity in HFTP.

In addition, the reform of HFTP also offers good opportunities for international pharmaceutical enterprises. China's total health spending reached ¥6.5196 trillion in 2019 (about US$1.0073 trillion based on the current exchange rate) (39), making it the world's second largest single-country health market. In the context of the COVID-19 pandemic, based on the latest outlook report of the World Bank, the growth of the world's major economies declined, yet China's economy was expected to grow by 8.5% in 2021 (40). This suggests that China would currently be one of the most vibrant economies in the world. Reform of HFTP not only provides a liberal and convenient licensed import channel for international MSE, but also significantly speeds up the official registration of licensed MSE in China by collecting and licensing RWD. Therefore, HFTP has become the best channel for global MSE to rapidly enter the huge Chinese market.

### HFTP's Medical Laws and Policy Reforms: Challenges

The reform of HFTP faces many challenges. In the current stage, medical tourism-associated public health risk is the major challenge facing the fields of medical and public health. HFTP has established nine new hospitals in Boao Lecheng, including two stem cell hospitals whose main business is anti-aging treatment (41). Due to the good rehabilitation environment of the tropical island in HFTP and the convenience of licensed MSE imported by new hospitals, HFTP is the most prosperous medical tourism market in China (42). However, the boom in medical tourism may introduce additional public health risks. So far, evidence suggests that the growing popularity of medical tourism in the USA has resulted in an increased infection rate of nontuberculous mycobacteria among its citizens (43). This suggests that the issue of additional public health risks should also be taken seriously by the authorities in HFTP. At present, the HFTP Law requires HFTP in Article 12 to strengthen public health security at ports (44). HFTP should make as soon as possible targeted law and policy for the additional public health risks that may increase from medical tourism.

Second, the acceleration of foreign MSE imports has also brought new challenges to the field of intellectual property management of HFTP. From 2012, China promised to implement stricter intellectual property protection, rapidly revise a series of laws and regulations such as the patent law, and establish new specialized intellectual property courts, one of which is located in HFTP (45) Thanks to these measures, HFTP's intellectual property management system has improved. However, the challenges faced by HFTP in the field of intellectual property management come from within, that is, a lack of sufficient professionals (46). In general, officers engaged in MSE intellectual property management should be familiar with relevant laws and policies of China's intellectual property management system and have a basic understanding and an appropriate level of professional knowledge (in, for example, biology or chemistry). This requires a relatively high level of education. However, Hainan is short of people with relevant higher education: as of 2020, only 13.92% of Hainan's total population had received a college education or above (47). Before the central government decided to establish HFTP, the lack of talents was not an urgent problem in Hainan. Hainan was only an island province dominated by tourism and its service industries, and the workload of intellectual property management was not large. Data disclosed by Hainan Province showed that by the end of November 2015, Hainan province had only 2,074 valid invention patents (48). In comparison, Huawei, a well-known technology company in China, had 87,805 patents in early 2018 (49). However, HFTP's reform will likely make Hainan the first choice for overseas MSE to enter the Chinese market. This implies that the number of MSE patent applications and intellectual property disputes in HFTP may surge. HFTP has realized that it is facing a key challenge: serious shortage of MSE intellectual property management talents (50). In response, HFTP has launched an unprecedented talent introduction plan, which aims to bring in on favorable terms more than a million people with professional and technical talents, including expertise in intellectual property management, from other regions at home and abroad (46). We will pay close attention to the effectiveness of the plan.

Third, the operation mode of shared hospital may have a negative impact on its diagnosis and treatment quality. A shared hospital provides a new hospital business model. It also means that a shared hospital will be composed of dozens of non-resident teams that do not necessarily relate easily to each other. For example, teams and hospitals will have less experience in daily cooperation and work docking than traditional hospitals. Unfamiliar cooperation and differences in work habits may lead to an amplification of avoidable medical mistakes. For example, in January 2021, a patient died while undergoing cochlear implantation surgery in a shared hospital. His family then pointed out that there were a series of avoidable low-level technical errors in the diagnosis and treatment process at the hospital, including the wrong recording of the patient's age and medical history (51). Perhaps because the shared hospital only provided a medical platform for resident teams, these errors were not found and corrected during the diagnosis and treatment stage. This tragedy suggests that HFTP needs to think swiftly about establishing a more effective supervision system for shared hospitals.

Fourth, the collection and application of RWD brings new regulatory challenges for HFTP in terms of data and information security. HFTP approved the reform measures of RWD, which have greatly improved the registration and listing speed of MSE. However, compared with the mature regulatory system of RCT in China, the regulatory system for RWD in HFTP is still not perfect. In terms of data and information security, HFTP requires all parties involved in RWD research to conduct security processing on data (especially data involving personal privacy) and establish a robust data security management system (52). However, these provisions may seem too principled and lack sufficient consideration of the details that may arise at the practical level. For example, the policy document of HFTP only requires the anonymization of relevant data, and does not specify who should be responsible for this activity in the study. Neither does it specify at which stage of the RWD study this step, which is critical to patient privacy, should be carried out. This means that researchers may have the opportunity to obtain from the hospital original data that has not been anonymized, which may increase the risk of patient privacy data disclosure. In addition, HFTP has not yet made detailed provisions on the legal liability of enterprises for RWD leakage, damage or loss due to accidents or other reasons. In short, HFTP urgently needs to further refine the relevant principled provisions on RWD.

Another concern is that the rapid introduction of foreign MSE may impact on local enterprises' R&D and production. Currently, there is still a big gap in MSE R&D and production capacity between Chinese enterprises and western counterparts (53). Although simplification of the procedures for the introduction and registration of foreign MSE by HFTP increases the accessibility of MSE, it also brings significant competition between local enterprises and large international enterprises. This should not be ignored. For example, since the marketing of the trastuzumab antibody drug conjugate DS8201 characterized by high cell membrane penetration and high portability in the USA in 2017, it has shown high efficacy in the posterior line treatment of a variety of tumors (such as advanced breast cancer) with overexpression or mutation of HER2 (54). However, in China it is only accessible in Hong Kong. For similar drugs that have been marketed in the mainland, the progression-free survival time of patients in posterior line treatment is inferior to that of patients taking DS8201: that is, 12.5 vs. 19.7 months in advanced breast cancer with the median treatment line of 6 (55). If DS8201 could enter the Chinese market quickly and at a low cost through the HFTP channels, the products produced by related enterprises of China would be very uncompetitive, and such enterprises would be deleteriously impacted.

However, we believe that HFTP's reform policy of accelerating the import process may have deepened the inferior position of Chinese enterprises in some areas, but this may be within the country's previous plans. At present, HFTP is mainly licensed to import MSE that is difficult for China to achieve domestic substitution in a short time. Most are specific drugs for the treatment of cancer and rare diseases. China Customs has previously reduced the tariff rate of these two types of MSE to 0% (56). It seems clear that China's tariff policy is the main reason why local companies face fierce competition in this field. HFTP's reform has not brought new adverse factors to the local pharmaceutical industry, but only intensified existing competition. Such difficult decisions are related to China's ambitious goal of raising the life expectancy of its 1.3 billion people by an additional year during the 14th Five-Year Plan period (2021–2025) (57). At a time when there is a significant difference between the efficacy of domestic MSE and imported MSE, China has had to temporarily sacrifice some interests of local enterprises in order to improve the drug accessibility for cancer and rare disease patients in order to improve per capita life expectancy. However, China has put forward the “Made in China 2025” strategic plan to help relevant companies further improve the level of MSE research and accelerate the process of localization of MSE, so as to support local companies currently disadvantaged (56). As a result, we believe that China still adheres to the policy of strengthening scientific and technological autonomy in the field of medicine and supporting domestic substitution. In this process, China hopes to reduce the medical burden of domestic patients through HFTP reforms in areas where domestic MSE is temporarily powerless. Although the impact of HFTP reform on local companies does not currently exceed China's expectations, considering the freer and more open development goals of HFTP, its special approval policy for imported MSE may be further expanded. The question remains: how to strike a reasonable balance between protecting production and R&D of local MSE and increasing accessibility of international MSE?

Overall, the reform of HFTP in the medical field is still in its infancy and faces many challenges. This paper has argued that HFTP's reforms in the medical field face more institutional challenges than reforms in various areas, including finance and taxation. This may relate to the lack of a mature domestic system template for HFTP's reform in this field. China has not yet formulated a systematic health law. The current health legal system is composed of more than 30 old and new laws. The lack of coordination between laws and sometimes conflicting situations still occurs, making it difficult to provide a systematic legal template for the reform of HFTP (58). In addition, HFTP law is mainly a principled authorization law that does not provide for more detailed provisions on the reform plans of HFTP. At present, the main roadmap of HFTP's reform is the *Overall Plan for the Construction of Hainan Free Trade Port* (OPCHFTP) issued by the central government, although OPCHFTP makes a few principled provisions, such as:

“We should support Hainan in introducing high quality foreign medical resources from abroad. We must draw from the trial experience of regional medical centers to explore and support the building of regional medical centers in Hainan” (59).

–Article 12, Section 1,Chapter III, OPCHFTP

In the absence of a systemic template, HFTP must try to construct a new institutional concept and framework for its medical reforms. While this implies that HFTP's reform may face additional unexpected challenges, as stated in OPCHETP, HFTP's reform experience and lessons in this field will still become an important reference for national reforms (59).

## Conclusion

HFTP is called “the pilot zone of national reform and opening up” by the Chinese government (60). This means that the successful experience of HFTP's reform is likely to be extended to other parts of China by the central government. Currently, the institutional reform of HFTP enables it to effectively increase the accessibility of imported MSE and the openness of the medical industry without excessive investment by the authorities. This may mean that China is probing how to further open up its huge medical market to improve its medical and public health standards. However, based on China's past reform experience, the often-cautious central government might consider carrying out similar reforms in other parts of China—but only after HFTP has both undertaken sufficient trial and error measures in relevant fields and improved the reform plan until it is seen to be economic, feasible and risk-controllable. As a result of such concerns, the reform of medical laws and policies in HFTP will necessarily be long-term and in-depth. This would not only bring major benefits for patients both in HFTP and elsewhere in China, but also provide an excellent opportunity for relevant international enterprises and institutions quickly to enter China's market. However, HFTP should form a systematic policy and legal framework in the health sector as soon as possible, and further improve supervision capabilities to ensure the stability and long-term nature of reform measures.

## Data Availability Statement

The original contributions presented in the study are included in the article/supplementary material, further inquiries can be directed to the corresponding author.

## Author Contributions

All authors made a significant contribution to this work, in relation to the conception, study design, execution, acquisition of data, analysis, and interpretation. All authors were involved in drafting, revising or critically reviewing the article, and gave final approval to the current version. Finally, all agreed on which journal the article should be submitted to and are accountable for all aspects of the work.

## Funding

This article is an outcome of the Hainan Provincial Philosophy and Social Science 2020 Planning Projects [Grant Number: HNSK(YB) 20-04].

## Conflict of Interest

FQ is a Fellow of the Royal Society of Arts (UK). YW is employed by Danzhou City Construction Investment Co., Ltd. QW is the Dean of Hainan University Law School. He is also a council member of the China Law Society, executive director of China Civil Procedure Law Research Institute, chairman of China Pacific Society Natural Resources Law Research Branch. The remaining author declares that the research was conducted in the absence of any commercial or financial relationships that could be construed as a potential conflict of interest.

## Publisher's Note

All claims expressed in this article are solely those of the authors and do not necessarily represent those of their affiliated organizations, or those of the publisher, the editors and the reviewers. Any product that may be evaluated in this article, or claim that may be made by its manufacturer, is not guaranteed or endorsed by the publisher.

## Abbreviations

MSE: medicine and sanitary equipment
RWD: real-world data
RWE: real-world evidence
B&R: the Belt and Road
HFTP: Hainan Free Trade Port
OPCHFTP: Overall Plan for the Construction of Hainan Free Trade Port
NMPA: National Medical Products Administration
NPC: National People's Congress
R&D: Research and development
RCT: randomized controlled trial
GIST: gastrointestinal stromal tumor.
